# Editorial: Proven and innovative methods of ex vivo examination of atherosclerotic and aortic valve lesions

**DOI:** 10.3389/fcvm.2024.1494278

**Published:** 2024-09-27

**Authors:** Michael Torzewski, Jianglin Fan, Anna Malashicheva

**Affiliations:** ^1^ Department of Laboratory Medicine and Hospital Hygiene, Robert Bosch Hospital, Stuttgart, Germany; ^2^ Guangdong Province Key Laboratory, Southern China Institute of Large Animal Models for Biomedicine, School of Pharmacy and Food Engineering, Wuyi University, Jiangmen, China; ^3^ Institute of Cytology, Almazov National Medical Research Centre, Saint-Petersburg, Russia

**Keywords:** atherosclerosis, innate & adaptive immunity, calcific aortic stenosis, medial arterial calcification, cohort analysis, animal model, atherosclerosis—diagnosis

**Editorial on the Research Topic**
Proven and innovative methods of ex vivo examination of atherosclerotic and aortic valve lesions

Unravelling the mysteries of atherosclerosis and its sequelae still remains a pressing issue due to the ongoing health and economic damage. Thereby, atherosclerosis research employs a diverse array of methodological approaches, each contributing unique insights into the disease's pathophysiology, risk factors, and potential treatments. Advanced sequencing techniques, imaging methods, multi-omics approaches, systems biology, machine learning, animal models, and large-scale epidemiological studies collectively enhance our understanding and management of atherosclerosis. These methodologies, when integrated, offer a comprehensive framework for advancing atherosclerosis research and improving clinical outcomes. The present research topic provides a cross section of them by drawing a bow from basic research over diagnostic tools to statistical approaches. The contributions are balanced and consist of three review articles and three original research contributions. The review “Molecular and cellular mechanisms of inflammation in atherosclerosis” by Popa-Fotea et al. provides a comprehensive overview of both innate and adaptive immunity being involved in the inflammatory component of atherosclerosis having the potential to open new therapeutic perspectives. However, since “the gods have placed the diagnosis before the therapy” (Carl Gustav Jung), and atherosclerosis remains asymptomatic for many years, the available diagnostic procedures hold fundamental importance. This is where the excellent review article by Poznyak et al. comes into play describing the most common and effective methods for diagnosing atherosclerosis by covering a wide range from structural and functional imaging of atherosclerosis to molecular methods and biomechanical analysis. The third pleasant review by Kim and Guzman addresses a rare but pathophysiologically intriguing entity, the medial arterial calcification (MAC). It particularly highlights the similarities and differences with atherosclerosis in terms of epidemiology, pathophysiology, and therapeutic approaches.

The keyword “calcification” serves as a bridge to now move to the original research contributions. First of all, Kachanova et al. present an innovative method with promising therapeutic implications matching perfectly our research topic title: a reproducible *ex vivo* tissue model for human aortic valve calcification. This experimental model includes the three-layered structure of the aortic valve, an architecture that is absent in almost all animal models and thus preserves interactions between cell populations and the extracellular matrix. The quality of the model is demonstrated using the specific Notch inhibitor Crenigacestat (LY30394478).

Next, Witzel et al. used the proven methodology of a sophisticated animal model demonstrating elegantly the impact of pituitary adenylate cyclase-activating polypeptide (PACAP), an anti-atherogenic neuropeptide on inflammatory processes and lipid homeostasis in different macrophage (MΦ) subtypes.

In another article, Peng et al. finally address one of the sequelae of atherosclerosis burden in the broadest sense by an age-period-cohort analysis of the 2019 Global Burden of Disease Study investigating differences in global, regional, and national time trends in disability-adjusted life years for atrial fibrillation and flutter (AF/AFL) The analysis shows that this disease plays a significant and increasing role worldwide withe the burden differing depending on socio-demographic index (SDI).

The present Research Topic will illustrate how methodology accounts for our understanding (and possibly misunderstanding) of the pathogenesis of two fundamental diseases with high impact on global health. It underscores the importance of comprehensive and critical assessments to clearly define the drawbacks and opportunities of the different methods and models. Alternatively, innovative new methodological approaches might broaden one's horizon with regard to fundamental questions regarding atherosclerosis and aortic valve sclerosis.

